# *Inter*- and *intra*-rater reliability for measurement of range of motion in joints included in three hypermobility assessment methods

**DOI:** 10.1186/s12891-018-2290-5

**Published:** 2018-10-17

**Authors:** Angela Schlager, Kerstin Ahlqvist, Eva Rasmussen-Barr, Elisabeth Krefting Bjelland, Ronnie Pingel, Christina Olsson, Lena Nilsson-Wikmar, Per Kristiansson

**Affiliations:** 1 Department of Public Health and Caring Sciences, Uppsala University, Husargatan 3, Box 564, 752 37 Uppsala, Sweden; 2 Karolinska Institutet, Department of Neurobiology, Care Sciences and Society, Division of Physiotherapy, Huddinge, Sweden; 3 Department of Obstetrics and Gynecology, Akershus University Hospital, Lørenskog, Norway; 4 Academic Primary Healthcare Centre, Stockholm County Council, Huddinge, Sweden

**Keywords:** Generalized joint hypermobility, *Inter*- and *intra*- reliability, Beighton score, Contompasis score, Hospital del mar criteria, Joint mobility assessment, Standardized protocol, Goniometer, Range of motion

## Abstract

**Background:**

Comparisons across studies of generalized joint hypermobility are often difficult since there are several classification methods and methodological differences in the performance exist. The Beighton score is most commonly used and has been tested for *inter-* and *intra*-rater reliability. The Contompasis score and the Hospital del Mar criteria have not yet been evaluated for reliability. The aim of this study was to investigate the *inter*- and *intra*-rater reliability for measurements of range of motion in joints included in these three hypermobility assessment methods using a structured protocol.

**Methods:**

The study was planned in accordance with guidelines for reporting reliability studies. Healthy adults were consecutively recruited (49 for *inter- *and 29 for *intra*-rater assessments). Intra-class correlations, two-way random effects model, (ICC 2.1) with 95% confidence intervals, standard error of measurement, percentage of agreement, Cohen’s Kappa (κ) and prevalence-adjusted bias-adjusted kappa were calculated for single-joint measured in degrees and for total scores.

**Results:**

The *inter-* and *intra*-rater reliability in total scores were ICC 2.1: 0.72–0.82 and 0.76–0.86 and for single-joint measurements in degrees 0.44–0.91 and 0.44–0.90, respectively. The difference between ratings was within 5 degrees in all but one joint. Standard error of measurement ranged from 1.0 to 6.9 degrees. The *inter-* and *intra*-rater reliability for prevalence of positive hypermobility findings the Cohen’s κ for total scores were 0.54–0.78 and 0.27–0.78 and in single joints 0.21–1.00 and 0.19–1.00, respectively. The prevalence- and bias adjusted Cohen’s κ, increased all but two values.

**Conclusions:**

Following a structured protocol, the *inter-* and *intra*-rater reliability was good-to-excellent for total scores and in all but two single joints, measured in degrees. The *inter-* and *intra*-rater reliability for prevalence of positive hypermobility findings was fair-to-almost perfect for total scores and slight-to-almost-perfect in single joints.

By using a structured protocol, we attempted to standardize the assessment of range of motion in clinical and in research settings. This standardization could be helpful in the first part of the process of standardizing the tests thus avoiding that assessment of GJH is based on chance.

**Electronic supplementary material:**

The online version of this article (10.1186/s12891-018-2290-5) contains supplementary material, which is available to authorized users.

## Background

Generalized joint hypermobility (GJH), defined as an increased range of motion (ROM) in several joints [1], is associated with longstanding musculoskeletal problems [2]. Many people with GJH seek primary care for pain and activity limitations [3, 4].

Joint ROM varies greatly in the general population [5, 6] and a joint ROM above two standard deviations from the average is suggested to be hypermobile [7]. The prevalence of GJH varies across gender, age, ethnicity and according to assessment methods and their cut-off points [8]. In Sweden, GJH is estimated to be present in approximately 10% of the general population [9].

Although GJH is an important criterion in the diagnosis of many heritable connective tissue disorders [3, 5] no agreed criteria exist [5, 10, 11]. Furthermore, which joints to include in diagnosing GJH has been debated [12]. The Beighton score (BeS) [13], which is a development of the Carter and Wilkinson score [14], is the most common diagnostic test for GJH worldwide [8, 15]. The BeS demonstrates good *inter*- and *intra*-rater reliability [15–17] but with conflicting evidence and methodological flaws [18]. Advantageously, the BeS is quick and easy to perform. However, the BeS only covers five joints particularly hinge joints and is an “all-or-none-test” with no indication regarding the degree of hypermobility [13]. Commonly used cut-off levels in the BeS vary between ≥4 and ≥ 5 for diagnosing GJH in adults [18].

Another assessment method is the Contompasis score (CS), a modification of the BeS which includes one additional joint. The CS is measured by grading the ROM and might be considered more time-consuming [19]. Furthermore, the Hospital del Mar criteria (HdM), which is a development of the Rotés-Querol, offer a wider view of joint mobility by assessing nine joints, including ball-and-socket-joints [12]. To our best knowledge, the *inter-* and *intra*-rater reliability of the CS and the HdM scores have not yet been evaluated.

Comparisons across studies of GJH assessments are hampered because a structured protocol is often lacking [20–23]. Neither the literature nor the criteria for diagnosis of GJH [3] and heritable connective tissue disorders [24] describes the test performances in detail [10, 18]. Although ROM measured in degrees using a goniometer has shown better *inter*-rater reliability, assessment of GJH is often based on visual assessment [15, 17, 25] with a dichotomous principle of judgement. The reliability is also affected by the joint structure, the level of pre-training and experience among the raters [26].

To identify people with GJH and subsequently tailor suitable interventions, reliable clinical assessment methods are important. Thus, there is a need for international consensus regarding performance, cut-off levels and interpretation of clinical assessments based on reliability studies of high quality [11, 18] to reduce the likelihood that the assessment of GJH is based on chance. Before deciding on the validity of these tests the reliability needs to be investigated in a standardized manner [18].

The aim of this study was to investigate the *inter*- and *intra*-rater reliability for measurements of ROM in joints included in three hypermobility assessment methods using a structured protocol.

## Methods

### Design

An *inter*- and *intra*-reliability study.

This study was planned and developed in accordance with “Guidelines for Reporting Reliability and Agreement Studies” (GRRAS) and “Quality Appraisal of Reliability Studies” (QAREL) [27, 28].

### Structured protocol and instruments

This study assessed *inter-* and *intra*-rater reliability of three hypermobility assessment methods, the BeS, the CS and the HdM for measuring joint ROM using a test-retest design which comprised in total 12 single joints. A protocol was developed to standardize the measurement of joint ROM (Additional file 1), which was further expanded from the original versions of the BeS, the CS and the HdM (Additional file 2). Starting position, positioning of the goniometer, anatomical landmarks, stabilization of adjacent structures and performances, using active or passive movement, were described and illustrated using photographs in the new protocol.

The BeS [13] comprises assessments of five joints, passive dorsiflexion of the fifth finger metacarpophalangeal joint, passive apposition of the thumb, passive hyperextension of the elbow and knee as well as forward flexion of the trunk*.* The first four joints are assessed bilaterally yielding a total score ranging from 0 to 9 [13]. The BeS scores of ≥4 and ≥ 5 points were used as cut-off levels for GJH.

The CS [19] comprises the assessment of six joints, which is similar to the BeS but with one additional joint, the foot flexibility test. Five joints are assessed bilaterally with each joint graded from two to six/or eight points with a total score range from 22 to 72 [19]. A cut-off level of ≥30 points for the CS was used to define GJH. The CS scores was modified because ROM in degrees for the elbow, knee and fifth finger were insufficiently graded and some degrees were represented in two score levels in the original description (Additional file 2).

The HdM [12] comprises the assessment of 10 items, passive apposition of the thumb, passive dorsiflexion of the fifth finger, passive hyperextension of the elbow, external shoulder rotation, hip abduction, patella hypermobility, ankle and foot hypermobility, first metatarsophalangeal joint, knee hyperflexion and easy bruising. Nine joints are assessed unilaterally on the non-dominant side. The last item deals with bruising; “Do you get bruises easily after minimal trauma?” Each hypermobile item scores one point, yielding a total score ranging from 0 to 10. The HdM ≥4 and ≥ 5 were set as cut-off levels for GJH [12]. The HdM measurement was modified by measuring passive opposition of the thumb with a goniometer instead of a ruler where < 15 degrees on the goniometer corresponds to < 21 mm on the ruler as used in the original description. Due to the lack of a reference value regarding a positive hypermobility finding for the ankle and the patella, ≥45 degrees was considered as hypermobile for the ankle [29, 30]. In addition, the measurement of the patella was standardized to make objective assessment possible (Additional file 1).

A goniometer (Medema Brodin, Kista Sweden, 31 cm or 21 cm with a 180° protractor and movable arms) was used. The small goniometer was used for measurements of the fifth finger and the big toe. Each joint was registered to the nearest 1-degree.

### Raters

Two physiotherapists, rater A (KA) and rater B (AS) assessed all of the participants. Both raters had clinical experience in the physical examination of patients with joint hypermobility attending primary care (27 and 24 years of experience respectively). To standardize the performance and to assure similar interpretations of the assessments, the two raters trained un-blinded on three occasions until consensus was reached, for a total of 24 h, before data collection. The training cohort included 21 persons.

### Participants

Information regarding the study was sent by e-mail to all 250 employees in a rehabilitation company within primary care in Stockholm, Sweden. The inclusion criteria were men and women aged between 18 and 65 years. For the *inter-*reliability study, we recruited the first consecutive 50 individuals who agreed to participate and who met the inclusion criteria. Of these, the first 30 participants were included in the *intra-*rater reliability study. Individuals with joint inflammatory signs, spasticity, joint-replacement, musculoskeletal injuries during the past 3 months and those who were not fluent in the Swedish language were excluded.

### Procedures

Self-reported sociodemographic data concerning gender, age and country of birth were obtained using a questionnaire. The raters examined the participants in separate examination rooms without the presence of other employees. The participants wore shorts and tank tops. No warming-up sessions were done before assessments. Reference dots were marked by the assessing rater on anatomical landmarks (Additional file 1), and were removed after each assessment session. The rater started each assessment with both oral and visual instructions about how the test would be performed.

The rater instructed the participant to stop the passive movement when they experienced that their joints were at an end-range position. The rater examined if it was possible to move the joint further without causing pain. In measurement of active ROM, the participant was asked: “Is this your maximum ROM?” For *inter*-raterreliability, the raters assessed the same participant with a minimum of 30 min and a maximum of 7 h between assessments. The raters were blinded with respect to each other’s results. To avoid recall bias in the *intra*-raterreliability study, rater B conducted the repeated assessments 7 to 14 days after the first assessment. The second assessment was performed at the same time of the day as the first.

A timetable assured that the time intervals between assessments were achieved and that the order in which raters assessed the participants varied. The order of the joint assessments changed every third assessment day for both *inter*- and *intra*-rater reliability examinations by starting from the end of the protocol (Additional file 1).

### Statistical analysis

Statistical analysis was conducted with R.3.3.1 (The R Project for Statistical Computing, Vienna, Austria). Intra-class correlations, two-way random effects model, ICC (2.1) with 95% confidence intervals (CI), were used to measure the *inter*- and *intra*-rater reliability for the quantitative measurements joint ROM (degrees) and total scores of the three hypermobility assessment methods [31]. The two-way models allow the error to be partitioned between systematic and random error [31, 32]. The ICC specific to the total score of the hypermobility assessment methods was used as the majority of these values were based on measured degrees. An ICC-score of < 0.40 was considered poor, 0.40–0.59 = fair/moderate, 0.60–0.74 = good and **≥** 0.75 = excellent [32]. The standard error of measurement (SEM) quantifies absolute reliability [33] and is referred to as the “typical” error [34]. The SEM was calculated using the residual mean square error from two-way repeated measures ANOVA. The SEM is important since a smaller SEM indicates more reliable results [33]. The value of an accepted SEM is a clinical decision.

For binary variables, the total percentage of agreement (P_a_) for prevalence of positive findings was calculated. To assess the proportion of agreement beyond that expected by chance Cohen’s Kappa (κ) was used [35]. A kappa value of κ = < 0.00 is considered as poor, 0.00–0.20 = slight, 0.21–0.40 = fair, 0.41–0.60 = moderate, 0.61–0.80 = substantial and ≥ 0.81almost perfect [36]. Since prevalence and bias affect the magnitude of the kappa coefficient, the prevalence-adjusted bias-adjusted kappa (PABAK) was calculated in addition to the obtained value of kappa [35].

With a significance level at 0.05 and a power of 80%, the sample size in this study was based upon an ICC score of at least 0.82 where a score of 0.6 or higher would be acceptable [37].

## Results

Forty-nine adults, 38 women and 11 men, mean (SD) age 39.8 (13.5) years participated in the *inter*-raterreliability study. Twenty-nine adults, 23 women and 6 men, mean (SD) age 39.9 (12.5) years participated in the *intra*-raterreliability study. The majority were Europeans, 96% and 97% respectively. One participant was excluded because of injury. The time interval from assessments in the *inter*-raterreliability study varied from 30 min to 7 h and between eight to 8 days in the *intra*-rater reliability study.

The *inter-* and *intra*rater-reliability for the total score of all assessment methods, using ICC 2.1, was good-to-excellent 0.72–0.82 and 0.76–0.86, respectively (Table 1).

**Table 1:** *Inter*-and *intra*-rater reliability of the total score of three hypermobility instruments

Variable	*Inter*-rater reliability					*Intra*-rater reliability				
Hypermobility instrument	Rater A mean (SD)	Rater B mean (SD)	*P*-value	Intra class correlation [1, 2] (95%Cl)	Standard error of measurement	Rating 1 mean (SD)	Rating 2 mean (SD)	*P*-value	Intra class correlation [1, 2] (95%Cl)	Standard error of measurement
BeS	1.4 (1.4)	1.3 (1.4)	0.59	0.72 (0.55–0.83)	0.7	1.4 (1.6)	1.1 (1.4)	0.11	0.76 (0.54–0.88)	0.7
HdM	2.7 (1.4)	2.6 (1.5)	0.42	0.81 (0.69–0.89)	0.6	2.5 (1.2)	2.3 (1.2)	0.08	0.86 (0.73–0.93)	0.4
CS	28.9 (4.3)	28.1 (4.1)	0.03	0.82 (0.69–0.89)	1.8	28.4 (3.5)	27.4 (3.8)	0.02	0.79 (0.57–0.90)	1.6

The hypermobility instruments used in this study were: *BeS* Beighton score, *CS* Contompasis score, *HdM* Hospital del Mar

The *inter-*rater reliability for measurements of joint ROM in degrees was good-to-excellent in all but three of the assessed joints (ICC 2.1: 0.67–0.91). For the hips and right calcaneus the reliability was moderate (ICC 2.1: 0.44–0.59). The differences between raters were within 5 degrees (0.1–4.3) in all but one measurement. The SEM ranged from 1.1 to 6.2 degrees (Table 2).

**Table 2:** *Inter*-rater reliability for measurement of joint mobility measured in degrees in three hypermobility instruments

Joint	Hypermobility instrument	Rater A mean (SD)	Rater B mean (SD)	Difference A-B	*P*-value	Intra class correlation [1, 2] (95%Cl)	Standard error of measurement
5th Finger, left	BeS, CS, HdM	76.9 (13.0)	73.4 (13.8)	3.5	< 0.001	0.85 (0.69–0.92)	4.7
5th Finger, right	BeS, CS, HdM	72.9 (14.6)	70.2 (14.0)	2.7	0.014	0.85 (0.74–0.92)	5.2
Thumb, left	BeS, CS, HdM	24.8 (11.0)	26.4 (10.9)	−1.6	0.017	0.91 (0.83–0.95)	3.2
Thumb, right	BeS, CS, HdM	27.4 (11.1)	27.3 (9.3)	0.1	0.859	0.89 (0.82–0.94)	3.4
Elbow, left	BeS, CS, HdM	5.6 (3.3)	4.4 (3.4)	1.2	0.002	0.69 (0.46–0.82)	1.8
Elbow, right	BeS, CS, HdM	5.4 (3.8)	4.2 (3.9)	1.2	0.005	0.67 (0.46–0.81)	2.1
Shoulder, left	HdM	62.1 (15.5)	60.3 (16.6)	1.8	0.081	0.90 (0.82–0.94)	5.0
Shoulder, right	HdM	63.4 (15.0)	61.4 (15.9)	2.0	0.054	0.89 (0.81–0.94)	5.1
Calcaneus, left	CS	3.2 (2.2)	2.3 (1.8)	0.8	< 0.001	0.68 (0.41–0.83)	1.0
Calcaneus, right	CS	2.9 (1.8)	2.3 (1.5)	0.6	0.011	0.59 (0.36–0.75)	1.0
Ankle, left	HdM	38.1 (5.5)	39.9 (6.2)	−1.8	0.001	0.77 (0.57–0.88)	2.6
Ankle, right	HdM	36.8 (6.2)	39.2 (6.2)	−2.4	< 0.001	0.82 (0.45–0.92)	2.2
Big toe, left	HdM	91.5 (10.9)	97.4 (14.3)	−5.9	< 0.001	0.73 (0.34–0.88)	5.5
Big toe, right	HdM	90.5 (12.2)	94.4(15.3)	−3.9	0.003	0.77 (0.59–0.87)	6.2
Knee extension, left	BeS, CS	3.4 (3.4)	4.0 (3.6)	−0.7	0.023	0.81 (0.68–0.89)	1.5
Knee extension, right	BeS, CS	3.5 (3.1)	4.3 (3.7)	−0.8	0.019	0.76 (0.60–0.86)	1.6
Hip abduction, left	HdM	35.8 (5.0)	32.4 (6.1)	3.4	< 0.001	0.44 (0.13–0.66)	3.8
Hip abduction, right	HdM	34.6 (6.6)	30.2 (5.8)	4.3	< 0.001	0.54 (0.08–0.77)	3.6

The hypermobility instruments used in this study were: *BeS* Beighton score, *CS* Contompasis score, *HdM* Hospital del Mar

The *intra*-rater reliability for measurements of joint ROM in degrees was good-to-excellent in all but three of the assessed joints (ICC 2.1: 0.60–0.90). For left hip and the calcaneus bilaterally the reliability was moderate (ICC 2.1: 0.44–0.51). The differences between test-retest assessments were within 3 degrees (0.0–2.7) in all but one of the measurements. SEM ranged from 1.0 to 5.7 degrees (Table 3).

**Table 3:** *Intra*-rater reliability for measurement of joint mobility measured in degrees in three hypermobility instruments

Joint	Hypermobility instrument	Rating 1 mean (SD)	Rating 2 mean (SD)	Difference 1–2	*P*-value	Intra class correlation [1, 2] (95%Cl)	Standard error of measurement
5th Finger, left	BeS, CS, HdM	71.5 (12.8)	71.6 (13.2)	−0.1	0.908	0.88 (0.77–0.94)	4.5
5th Finger, right	BeS, CS, HdM	71.6 (13.1)	70.4 (12.9)	1.2	0.312	0.88 (0.75–0.94)	4.6
Thumb, left	BeS, CS, HdM	26.3 (8.7)	27.3 (8.9)	−1.0	0.207	0.89 (0.78–0.94)	3.0
Thumb, right	BeS, CS, HdM	28.6 (9.4)	28.6 (8.3)	0.0	1.000	0.90 (0.79–0.95)	2.8
Elbow, left	BeS, CS, HdM	4.8 (4.0)	5.2 (3.5)	−0.4	0.551	0.60 (0.30–0.79)	2.4
Elbow, right	BeS, CS, HdM	5.4 (4.1)	4.2 (4.2)	1.2	0.040	0.71 (0.47–0.86)	2.1
Shoulder, left	HdM	60.4 (15.5)	62.5 (15.6)	−2.1	0.175	0.86 (0.73–0.93)	5.7
Shoulder, right	HdM	61.5 (13.9)	63.0 (15.7)	−1.5	0.288	0.87 (0.74–0.94)	5.3
Calcaneus, left	CS	2.4 (1.5)	1.9 (1.5)	0.5	0.105	0.44 (0.11–0.69)	1.1
Calcaneus, right	CS	2.6 (1.3)	2.4 (1.5)	0.2	0.432	0.51 (0.19–0.74)	1.0
Ankle, left	HdM	40.2 (5.1)	40.0 (6.2)	0.3	0.676	0.81 (0.64–0.91)	2.5
Ankle, right	HdM	39.6 (6.8)	39.6 (6.0)	0.0	0.959	0.85 (0.70–0.93)	2.5
Big toe, left	HdM	99.9 (13.4)	93.5 (14.6)	6.3	< 0.001	0.79 (0.31–0.92)	5.2
Big toe, right	HdM	93.1 (14.8)	90.4 (16.5)	2.7	0.079	0.86 (0.72–0.93)	5.6
Knee extension, left	BeS, CS	4.4 (3.5)	3.9 (3.6)	0.5	0.285	0.77 (0.58–0.89)	1.7
Knee extension, right	BeS, CS	4.8 (3.4)	4.4 (4.1)	0.4	0.515	0.66 (0.40–0.83)	2.2
Hip abduction, left	HdM	30.9 (5.0)	33.4 (5.0)	−2.5	0.010	0.45 (0.11–0.70)	3.6
Hip abduction, right	HdM	28.8 (5.9)	30.5 (6.2)	−1.7	0.062	0.67 (0.40–0.83)	3.4

The hypermobility instruments used in this study were: *BeS* Beighton score, *CS* Contompasis score, *HdM* Hospital del Mar

For *inter-*rater reliability, the agreement (P_a)_ for the prevalence of positive hypermobility findings ranged from 80 to 98% for all total scores and Cohen’s (κ) was moderate-to-substantial (κ = ≥0.54–0.78). The PABAK increased the results (κ = ≥0.59–0.96), (Table 4). Regarding prevalence of positive hypermobility findings for separate joint assessments, the P_a_ ranged from 80 to 100%, except for the calcaneus. Cohen’s (κ) was substantial-to-almost perfect for 13 of the 21 joint assessments (κ = 0.63–1.00) while the PABAK was substantial-to-almost perfect in all but three joint assessment (κ = 0.63–1.00), (Table 4).

**Table 4:** *Inter*-rater reliability for prevalence of positive hypermobility findings for total score and for single-joints

*Total score*/single joint	Prevalence of positive findings		Agreement	Reliability		Prevalence Index	Bias Index
	Rater A	Rater B	Prevalence of positive findings (%)				
	*n* (%)	*n* (%)		Kappa (95% Cl)	Prevalence-adjusted bias-adjusted kappa-value (95% Cl)		
*BS ≥ 4*	3 (6)	4 (8)	94	0.54 (0.26–0.82)	0.88 (0.66–0.97)	0.86	0.02
*BS ≥ 5*	2 (4)	1 (2)	98	0.66 (0.39–0.92)	0.96 (0.78–1.00)	0.94	−0.02
*HdM ≥ 4*	13 (27)	11 (22)	92	0.78 (0.50–1.06)	0.84 (0.61–0.95)	0.51	0.04
*HdM ≥ 5*	7 (14)	8 (16)	94	0.76 (0.48–1.04)	0.88 (0.66–0.97)	0.69	0.02
*CS ≥ 30*	18 (37)	14 (29)	80	0.54 (0.26–0.81)	0.59 (0.31–0.80)	0.35	−0.08
5th Finger, left	11 (22)	7 (14)	92	0.73 (0.46–1.00)	0.84 (0.61–0.95)	0.63	0.08
5th Finger, right	7 (14)	4 (8)	94	0.70 (0.43–0.96)	0.88 (0.66–0.97)	0.78	−0.06
Thumb, left	11 (22)	11 (22)	100	1.00 (0.72–1.28)	1.00 (0.79–1.00)	0.55	0.00
Thumb, right	7 (14)	6 (12)	98	0.91 (0.63–1.19)	0.96 (0.78–1.00)	0.73	−0.02
Elbow, left	8 (16)	5 (10)	82	0.21 (−0.06–0.48)	0.63 (0.36–0.82)	0.73	−0.06
Elbow, right	7 (14)	7 (14)	88	0.50 (0.22–0.78)	0.76 (0.50–0.91)	0.71	0.00
Shoulder, left	1 (2)	2 (4)	98	0.66 (0.39–0.92)	0.96 (0.78–1.00)	0.94	0.02
Shoulder, right	5 (10)	4 (8)	94	0.63 (0.36–0.91)	0.88 (0.66–0.97)	0.82	−0.02
Calcaneus, left	30 (61)	22 (45)	76	0.52 (0.26–0.79)	0.51 (0.22–0.73)	− 0.06	0.16
Calcaneus, right	28 (57)	19 (39)	73	0.49 (0.22–0.75)	0.47 (0.18–0.70)	0.04	−0.18
Ankle, left	7 (14)	11 (22)	80	0.33 (0.06–0.60)	0.59 (0.31–0.80)	0.63	0.08
Ankle, right	6 (12)	11 (22)	86	0.51 (0.25–0.77)	0.71 (0.46–0.88)	0.65	0.10
Big toe, left	31 (63)	35 (71)	84	0.63 (0.36–0.91)	0.67 (0.41–0.85)	−0.35	0.08
Big toe, right	28 (57)	32 (65)	84	0.66 (0.38–0.93)	0.67 (0.41–0.85)	−0.22	0.08
Knee extension, left	3 (6)	6 (12)	90	0.40 (0.13–0.66)	0.80 (0.56–0.93)	0.82	0.06
Knee extension, right	1 (2)	5 (10)	92	0.31 (0.11–0.51)	0.84 (0.61–0.95)	0.88	0.08
Knee flexion, left	39 (80)	37 (76)	92	0.77 (0.49–1.04)	0.84 (0.61–0.95)	−0.55	− 0.04
Knee flexion, right	38 (78)	36 (73)	92	0.78 (0.50–1.06)	0.84 (0.61–0.95)	− 0.51	− 0.04
Trunk flexion	12 (24)	11 (22)	98	0.94 (0.66–1.22)	0.96 (0.78–1.00)	0.53	−0.02
Patella, left	4 (8)	4 (8)	100	1.00 (0.72–1.28)	1.00 (0.79–1.00)	0.84	0.00
Patella, right	4 (8)	5 (10)	98	0.88 (0.60–1.16)	0.96 (0.78–1.00)	0.82	0.02
Hip abduction, left	0 (0)	0 (0)	NA	NA	NA		
Hip abduction, right	0 (0)	0 (0)	NA	NA	NA		

The hypermobility instruments used in this study were: *BS* Beighton score, *CS* Contompasis score, *HdM* Hospital del Mar, *NA* Not applicable, none of the participants reached the cut off limit

For *intra-*rater reliability, the P_a_ for prevalence of positive hypermobility findings ranged from 72 to 97% for all total assessment scores. Cohen’s (κ) was fair-to-substantial (κ = 0.27–0.78) and the PABAK was moderate-to-almost perfect (κ = 0.45–0.93), (Table 5). For prevalence of positive hypermobility findings regarding single joint assessments, the P_a_ ranged from 79 to 100% excpept for the calcaneus. Cohen’s (κ) was substantial-to-almost perfect in 13 of the 21 joint assessments (κ = 0.61–1.00). The PABAK was substantial-to-almost perfect in all but three joint assessment (κ = 0.66–1.00), (Table 5).

**Table 5:** *Intra*-rater reliability for prevalence of positive hypermobility findings for total score and for single-joints

*Total score*/single joint	Prevalence of positive findings		Agreement	Reliability		Prevalence index	Bias index
	Rater A	Rater B	Prevalence of positive findings (%)	Kappa (95% Cl)	Prevalence-adjusted bias-adjusted kappa-value (95% Cl)		
	*n* (%)	*n* (%)					
*BeS ≥ 4*	3 (10)	2 (7)	97	0.78 (0.43–1.14)	0.93 (0.64–1.00)	0.83	−0.03
*BeS ≥ 5*	3 (10)	1 (3)	93	0.47 (0.16–0.78)	0.86 (0.54–0.98)	0.86	−0.07
*HdM ≥ 4*	7 (24)	4 (14)	90	0.67 (0.33–1.01)	0.79 (0.45–0.96)	0.62	−0.10
*HdM ≥ 5*	2 (7)	3 (10)	97	0.78 (0.43–1.14)	0.93 (0.64–1.00)	0.83	0.03
*CS ≥ 30*	9 (31)	5 (17)	72	0.27 (−0.07–0.60)	0.45 (0.06–0.75)	0.52	−0.14
5th Finger, left	3 (10)	3 (10)	100	1.00 (0.64–1.36)	1.00 (0.66–1.00)	0.79	0.00
5th Finger, right	3 (10)	2 (7)	97	0.78 (0.43–1.14)	0.93 (0.64–1.00)	0.83	−0.03
Thumb, left	8 (28)	5 (17)	90	0.71 (0.36–1.06)	0.79 (0.45–0.96)	0.55	−0.10
Thumb, right	5 (17)	2 (7)	90	0.52 (0.20–0.84)	0.79 (0.45–0.96)	0.76	−0.10
Elbow, left	5 (17)	4 (14)	90	0.61 (0.25–0.97)	0.79 (0.45–0.96)	0.69	−0.03
Elbow, right	6 (21)	4 (14)	93	0.76 (0.41–1.11)	0.86 (0.54–0.98)	0.66	−0.07
Shoulder, left	1 (3)	1 (3)	100	1.00 (0.64–1.36)	1.00 (0.66–1.00)	0.93	0.00
Shoulder, right	2 (7)	3 (10)	90	0.35 (−0.01–0.70)	0.79 (0.45–0.96)	0.83	0.03
Calcaneus, left	14 (48)	10 (34)	66	0.30 (−0.05–0.65)	0.31 (−0.09–0.64)	0.17	− 0.14
Calcaneus, right	13 (45)	10 (34)	76	0.50 (0.15–0.86)	0.52 (0.13–0.79)	0.21	−0.10
Ankle, left	5 (17)	6 (21)	90	0.66 (0.30–1.03)	0.79 (0.45–0.96)	0.62	0.03
Ankle, right	7 (24)	6 (21)	83	0.51 (0.14–0.87)	0.66 (0.28–0.88)	0.55	−0.03
Big toe, left	24 (83)	18 (62)	79	0.51 (0.19–0.83)	0.59 (0.21–0.84)	−0.45	−0.21
Big toe, right	15 (52)	16 (55)	83	0.65 (0.29–1.02)	0.66 (0.28–0.88)	−0.07	0.03
Knee extension, left	4 (14)	3 (10)	97	0.84 (0.48–1.20)	0.93 (0.64–1.00)	0.76	−0.03
Knee extension, right	3 (10)	4 (14)	83	0.19 (−0.17–0.55)	0.66 (0.28–0.88)	0.76	0.03
Knee flexion, left	21 (72)	22 (76)	90	0.73 (0.37–1.09)	0.79 (0.45–0.96)	−0.48	0.03
Knee flexion, right	22 (76)	23 (79)	97	0.90 (0.54–1.26)	0.93 (0.64–1.00)	−0.55	0.03
Trunk flexion	6 (21)	6 (21)	100	1.00 (0.64–1.36)	1.00 (0.66–1.00)	0.59	0.00
Patella, left	2 (7)	3 (10)	97	0.78 (0.43–1.14)	0.93 (0.64–1.00)	0.83	0.03
Patella, right	2 (7)	5 (17)	90	0.52 (0.20–0.84)	0.79 (0.45–0.96)	0.76	0.10
Hip abduction, left	0 (0)	0 (0)	NA	NA	NA		
Hip abduction, right	0 (0)	0 (0)	NA	NA	NA		

The hypermobility instruments used in this study were: *BeS* Beighton score, *CS* Contompasis score, *HdM* Hospital del Mar, *NA* Not applicable, none of the participants reached the cut off limit

*The inter-* and *intra-*rater reliability for the prevalence of positive hypermobility findings for the hip- abduction are not reported since none of the participants reached the cut off limit of > 85 degrees (Tables 4 and 5).

## Discussion

To the best of our knowledge, this is the first study to investigate the *inter*- and *intra*-rater reliability of the Beighton score, the Contompasis score and the Hospital del Mar criteria. We used a structured protocol including descriptions of testing positions, starting positions, goniometer positions, anatomical landmarks, stabilization of adjacent structures and performance illustrated by photos.

Following this structured protocol with use of a goniometer, all of the three hypermobility assessment methods, the BeS, the CS and the HdM, showed good-to-excellent *inter*- and *intra*-rater reliability for the total scores and for the majority of the single-joint measurements in degrees. The SEM for *inter*- and *intra*-rater reliability ranged from 1.0 to 6.2 degrees.

Previous reliability studies of the BeS using a protocol have presented similar results to those in this study [12, 15–17, 25, 38, 39]. However, comparisons with these studies are complicated as the testing procedures vary. This will affect the measurement of joint ROM [40] and thus influence the results [10]. In addition, many studies reported the use of no [21, 22] or an insufficient protocol [23, 25, 41, 42]. Comparisons are further hampered due to differences regarding the use or lack of use of a goniometer, reference lines for the goniometer and for anatomical landmarks, insufficient stabilization of adjacent structures, active or passive testing, testing positions, cut-off levels and statistical methods [15–17, 21, 22, 25, 38, 39, 41, 42].

To the best of our knowledge, this was the first *inter-* and *intra*-rater reliability study of the CS and the HdM and the first reliability study using measurement in degrees for joints included in the three hypermobility assessment methods. The *inter*- and *intra*-rater reliability was good-to-excellent for the majority of the single-joint assessments. Since prevalence and bias affect the magnitude of Cohen’s (κ), it is recommended to also calculate the PABAK [35]. Due to adjusting for prevalece and bias, higher PABAK than Cohen’s (κ) was found across all the results (Tables 4 and 5).

The difference between and within the raters in the present study was less than five degrees in all but one measurement which is in accordance with other studies [38, 43]. This is within an acceptable measure, as a variation of ±5 degrees in goniometric measurements is generally accepted in the clinic [44, 45].

The *inter*-and *intra*-rater reliability was moderate for some joints, indicating difficulties in the performance of these assessments. Joints without ROM end points, such as the elbow, the fifth finger and the knee might be considered more challenging to measure. This could be the reason why these joints in the BeS showed the lowest kappa values and the lowest P_a_ for the prevalence of positive hypermobility findings in this study and as well as in other studies [15, 17, 25, 42]. We stabilized the wrist and the fourth finger when measuring the fifth finger ROM since the test phase showed an increased ROM when the adjacent structures were not stabilized. This may affect the prevalence. Therefore, there is a need for consensus in the performance.

We have not found any documentation regarding the selection of joints for the criteria of the GJH.

In addition to study reliability of the BeS with a structured protocol, this study also aimed to establish the *inter*- and *intra*-rater reliability for the measurement of ROM in joints other than those included in the BeS. Children with joint hypermobility assessed with the BeS were equally hypermobile in their ball-and-socket-joints [43]. Thus, the importance of ball-and-socket-joints in adults with GJH requires further study.

Following this structured protocol with standardized assessments provided an excellent *inter*- and *intra-*rater reliability for the measurement of external rotation of the shoulder ICC 2.1: 0.89–0.90 and 0.86–0.87, respectively.

In accordance with another study [15], we reported low *inter*- and *intra*-rater reliability in measurements of hip-abduction, which may be due to insufficient stabilization of the pelvis. Furthermore, as in the hip-abduction measurement of elbow and calcaneus showed wide confidence intervals. The lack of precision in these measurements, as displayed by the wide CIs, suggests that the reliability should be interpreted with care. For the elbow, this could depend on a large valgus angle that falsely might give an impression of hypermobility [17]. Moreover, it is difficult to evaluate the reliability of the calcaneus tilt since the ROM is within the measurement error of the goniometer. This finding suggests that the calcaneus tilt should be excluded in the assessment of GJH. Other disputable tests included in the HdM are the knee-hyperflexion and the big toe-extension test. Most participants scored positive on these tests even though they were not hypermobile in other joints, suggesting that the risk of a false positive finding in the general population is high. Despite good-to-excellent *inter*- and *intra*-rater reliability, these tests are not adequate to identify joint hypermobility, as also confirmed in another study [23]. We therefore propose that these tests should be removed from the HdM. The remarkably high prevalence of positive hypermobility findings for knee-flexion and big toe-extension may have resulted in a higher prevalence of hypermobility in the HdM compared to the BeS in this study.

There was a difference in big toe-extension between right and left side for both *inter*- and* intra*-rater reliability, indicating a systematic error. This may be explained by the fact that both raters were right-handed.

None of the participants had hypermobile hip-abduction and few had hypermobile external rotation of the shoulder even though measurements showed hypermobility in other joints. This may indicate that the cut-off value for hypermobility in these joints is too high in the HdM. A too high cut-off value increases the risk of underdiagnosing a possible hypermobility. In accordance with another study [15] cut-off levels for hypermobility above 55 degrees for hip-abduction [30, 46] and above 68 degrees for the shoulder external rotation [46] are supported.

We defined cut-off levels for the three hypermobility assessment methods. A cut-off level of the CS ≥ 30 for GJH was used in this study which corresponds to the BeS cut-off level of ≥4 points [47]. Previous reliability studies concerning the CS also used other cut-off levels [47, 48] than in the original description [19]. A cut-off level of ≥30 for the CS had a lower kappa value compared to a cut-off level of ≥4 or ≥ 5 when using the BeS and the HdM in this study. This may be due to the fine-scale grading of the CS, suggesting that the CS is more sensitive to measurement differences. Another possible explanation could be the small ROM of the calcaneus tilt and the cut-off levels for hypermobility making the judgement less reliable as mentioned above.

The strength of this study is that it was planned and developed in accordance with GRRAS [27] and QAREL [28]. It included a structured protocol with use of size-adjusted goniometers and a comprehensive description of the procedures for performing the assessments illustrated by photographs as recommended [18]. Two experienced physiotherapists, who had trained before the study, performed the measurements. The experience of the rater is important [15] as confirmed in another study showing that *inter*-rater variability increased as the level of medical education decreased [42]. Furthermore, the stability of joint ROM was taken into account for time intervals of assessments.

The raters stabilized adjacent structures to reduce the risk of false positive hypermobility findings and mainly used passive tests to assure that the end-range position was reached, since passive ROM is greater than active [30].

This study described testing positions since this impact the ROM and an optimal position should facilitate reaching the end-range position. Testing position of adjacent joints is also important. For example, the position of the wrist and the elbow will impact the ROM of the thumb and the fifth finger [13, 38, 39].

A limitation in the present study is that the degree of agreement set at 80% in the training phase was not specified as recommended by “The International Federation for Manual/Musculoskeletal Medicine” (FIMM) [49]. The rater only measured each subject once to imitate clinical practice. Additionally, another study reported that mobility of joints increased significantly in consecutive measurements [38]. Furthermore, our aim was to measure the participant at the same time point at all testing occasions as it might be important to take this into consideration. However, about half of the participants were not assessed at the same time of the day. This may have influenced the results.

Since both raters were experienced, the use of a third, less experienced rater might have increased the generalizability in a clinical context. However, the generalizability also depends on the raters´ ability to follow the testing procedures in a structured protocol. In our study, the raters were experienced. Still, the reliability was not excellent for all measures. For instance, a ROM measurement close to the cut off level for a positive hypermobility finding could be interpreted as positive by one rater and negative by the other. Future implementation of new tools to measure ROM will hopefully increase the accuracy.

The choice of using a general population instead of a population diagnosed with GJH might be considered a limitation. However, our main focus was to standardize assessment of joint ROM in degrees, regardless whether the participant was hypermobile or not. The decision to measure joint ROM in a general population with an expected variation in joint mobility aimed to generalize our result to a broader context.

If joint hypermobility is suspected after screening in clinical practice, a standardized joint assessment should be performed for diagnosing GJH. Moreover, to be able to compare GJH studies and to reach international consensus regarding diagnosing GJH-related disorders, a description with standardization of procedures for performing assessments of ROM is needed [18].

## Conclusions

The *inter-* and *intra*-rater reliability for total scores was good-to-excellent for the BeS, the CS and the HdM following a structured protocol. However, the *inter-* and *intra-*rater reliability was poor-to-moderate in some single joint measurements, indicating difficulties in the performance of these tests. This study includes a structured protocol with a comprehensive description of the performance of joint mobility measurement in several joints. This could be helpful in the first part of the process of standardizing the tests. Standardization for measurement of GJH is needed to provide that the criteria for assessment of GJH is not based on chance and may contribute to minimizing the risk of scoring healthy individuals with GJH. Future studies of reliability and validity should use a standardized protocol to assess persons with GJH. Furthermore, joint measurements other than those included in the BeS are needed, particularly tests assessing ball-and-socket-joints to consider whether these joints are important when diagnosing GJH. In addition, the study indicates that the cut-off value for hypermobility is too high in some joints and needs to be further studied.

## Additional files

Additional file 1: Structured protocol for measurement of range of motion in joints included in the Beighton score, the Contompasis score and the Hospital del Mar Criteria. (DOCX 1226 kb)

Additional file 2: Original description of the hypermobility instruments, the Beighton score, the Contompasis score and the Hospital del Mar Criteria. (DOCX 20 kb)

## Abbreviations

ANOVA: analysis of variance
BeS: Beighton score
CI: Confidence interval
FIMM: International Federation for Manual/Musculoskeletal Medicine
GJH: Generalized joint hypermobility
GRRAS: Guidelines for Reporting Reliability and Agreement Studies
HdM: Hospital del Mar criteria
ICC: Intra class correlation
P_a_: Total percentage of agreement
PABAK: Prevalence-adjusted bias-adjusted kappa
QAREL: Quality Appraisal of Reliability Studies
ROM: Range of motion
SD: Standard deviation
SEM: Standard error of measurement
κ: Kappa
