# Supplementary material for: Inter- and intra-rater reliability for measurement of range of motion in joints included in three hypermobility assessment methods
Source: BMC Musculoskelet Disord. 2018 Oct 17;19:376. doi: 10.1186/s12891-018-2290-5 (PMC6192271; doi:10.1186/s12891-018-2290-5)
Supplement: Supplementary file 1 — Structured protocol for measurement of range of motion in joints included in the Beighton score, the Contompasis score and the Hospital del Mar Criteria. (DOCX 1226 kb) [file 12891_2018_2290_MOESM1_ESM.docx]

***Additional file 1***

Structured protocol for clinical tests in Beighton score, Contompasis score and Hospital del Mar criteria

Performer_____________________ Date____________________

Measurement occasion:

1:1🞎 1:2🞎 2🞎

Name_________________________

TESTS IN SITTING POSITION

**Passive dorsiflexion of the fifth metacarpophalangeal joint (2, 3, 4)**

Starting position: The participant sits with the entire forearm resting on a table with the palm down and the elbow flexed.

Goniometer position and anatomical landmarks: Place the center of the goniometer over the fifth metacarpophalangeal joint. The stationary arm of the goniometer is placed on the table parallel to the longitudinal axis of the radius, with the protractor of the goniometer facing up. Place the movable arm of the goniometer parallel to the longitudinal axis of the proximal phalanx of the fifth finger.

Stabilization: Stabilize the proximal third finger and the wrist so that no movement occurs in these joints.

Performance: The assessor will slowly extend the fifth finger passively to the limit of motion until resistance is elicited without the other fingers, the distal joint of the fifth finger or wrist moves. Use a small goniometer.


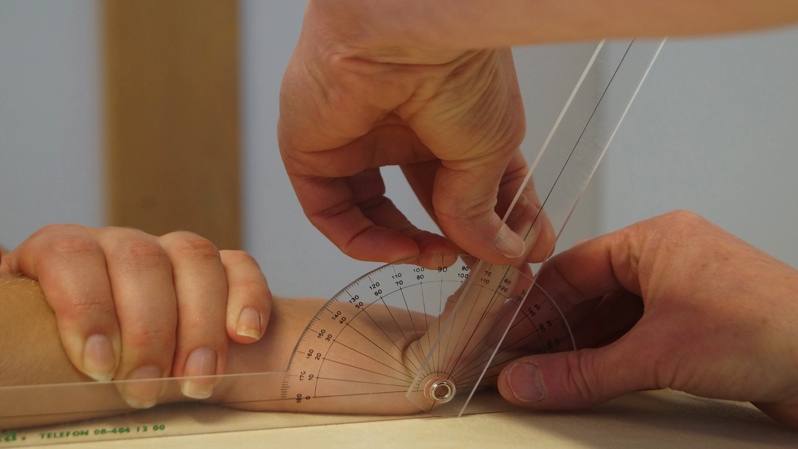
Left finger hyperextension

🞎 Left, no

🞎 Left, yes

Right finger hyperextension

🞎 Right, no

🞎 Right, yes

Left fifth finger angle: ______

Right fifth finger angle: ______

**Passive apposition of the thumb to the flexor side of the forearm (2, 3, 4)**

Starting position: The participant sits with the elbow flexed, leaning the elbow on a table. Mark reference dot on the lateral epicondyle

Goniometer position and anatomical landmarks: Place the center of the goniometer in the level of radio carpal joint. Keep the stationary arm of the goniometer parallel to the longitudinal axis of the radius towards the lateral epicondyle with the protractor of the goniometer towards the thumb. The movable arm of the goniometer is placed towards the tip of the thumb.

Stabilization: None

Performance: The participant flexes the wrist passively to fully supinated forearm (volar flexion of the wrist) with her other hand and moves the thumb, holding at the distal interphalangeal joint, in a direction towards the flexor side of the forearm, to the end of the movement. After that the assessor will slowly push the thumb forward to ensure that the limit of motion is gained. Use a large goniometer.


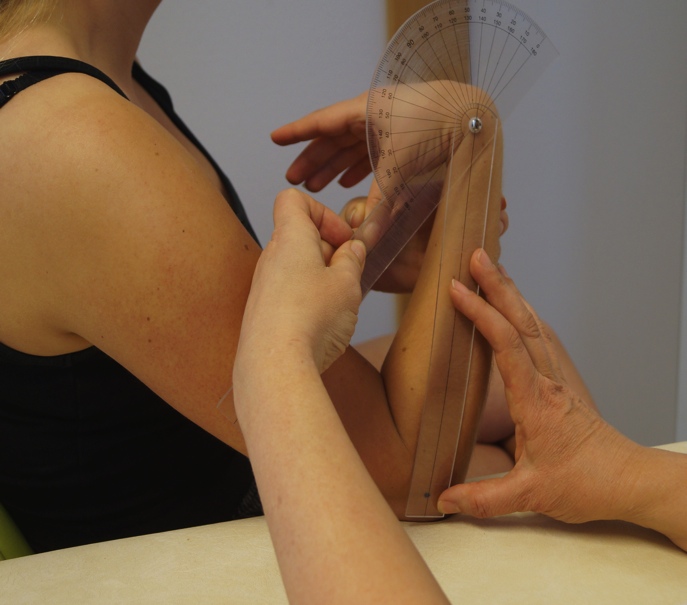


Left

🞎 No touch of thumb to forearm

🞎 Thumb touches forearm

🞎 Thumb digs into forearm easily

🞎 Thumb overlaps the outside of forearm

Right

🞎 No touch of thumb to forearm

🞎 Thumb touches forearm

🞎 Thumb digs into forearm easily

🞎 Thumb overlaps the outside of forearm

Left thumb angle: ______

Right thumb angle: ______

**Active external rotation of the shoulder (4)**

Starting position: The participant sits with the upper arms adducted close to the body with elbows flexed to 90 degrees, with the forearm in mid position thumbs facing up. The forearm is parallel to the sagittal plane.

Goniometer position and anatomical landmarks*:* Place the center of the goniometer above the middle of the acromioclavicular joint with the protractor of the goniometer outwards and the stationary arm of the goniometer pointing straight forward. Move the movable arm of the goniometer parallel to the longitudinal axis of the radius.


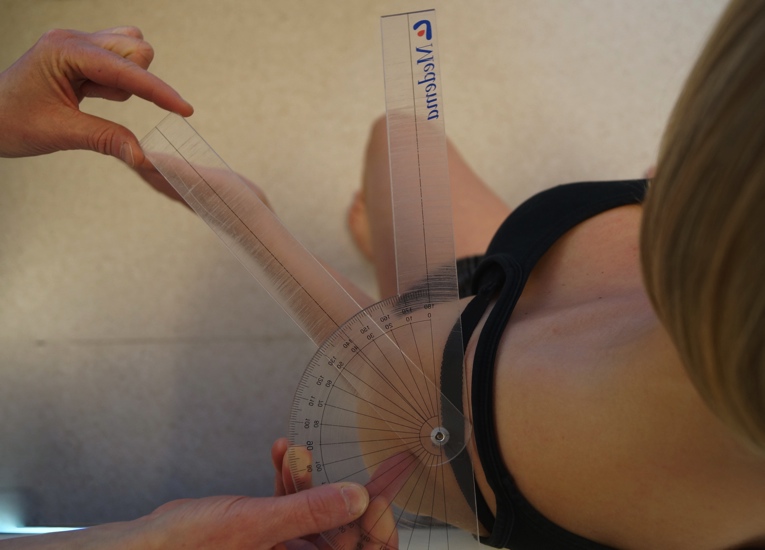
Stabilization: None

Performance*:* The participant actively moves both the forearms outwards, without dropping the adduction to the side of the body, to the outer position. The participant is then asked “is this your maximum mobility”. Use a large goniometer.

Left shoulder angle: _______

Right shoulder angle: ______

**Passive hyperextension of the elbow (1, 2, 3, 4)**

Starting position: The participant sits with the arm along the body and gently adducted to the side. The forearm is in full supination with the biceps brachii pointing forward in order to neutralize the valgus angle of the elbow. Mark reference dots on the middle of acromion and processus styloideus radii.

Goniometer position and anatomical landmarks: Place the center of the goniometer over the lateral epicondyle of the humerus. Place the stationary arm of the goniometer, parallel to the longitudinal axis of the humerus, pointing toward the middle of the acromion with the protractor of the goniometer faced backwards and hold firmly. Place the movable arm of the goniometer parallel to the longitudinal axis of the radius, pointing toward the styloid process of the radius.

Stabilization: Make sure that no the shoulder is stable with the upper hand of the assessor.


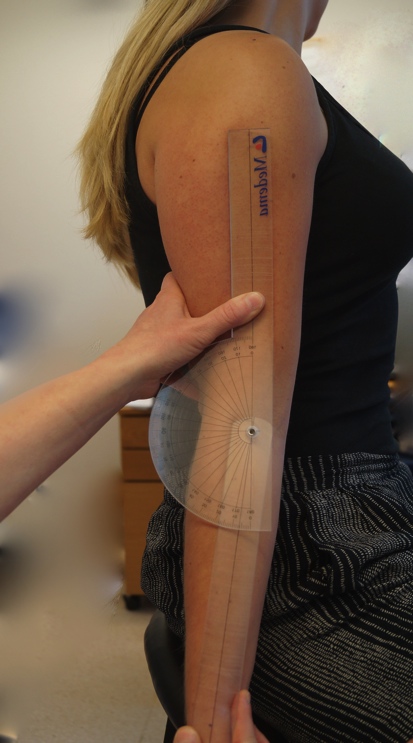
Performance: The participant will first actively extend the elbow to its maximum. Then the assessor will slowly push the forearm passively in further extension to ensure that the limit of motion is gained. Use a large goniometer.

Left elbow hyperextension:

🞎 No

🞎 Yes

Right elbow hyperextension:

🞎 No

🞎 Yes

Left elbow angle: ______

Right elbow angle: ______

TESTS IN STANDING POSITION

**Active forward flexion of the trunk, with straight knees and palms resting on the floor (2, 3, 4)**

Starting position: The participant stands with straight knees and groin width distance between feet.

Goniometer position and anatomical landmarks*:* None

Stabilization: None


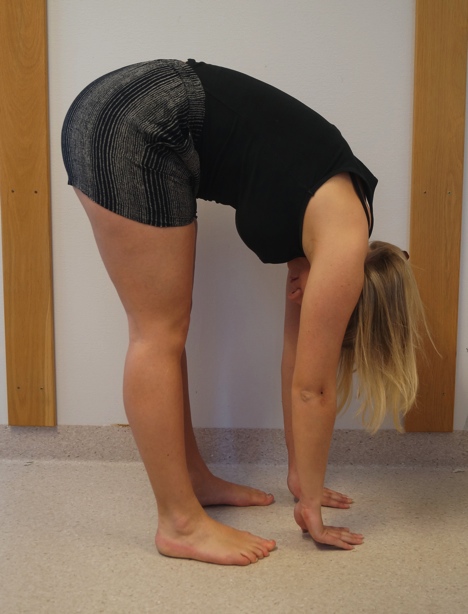
Performance: The participant flexes the trunk actively forward with the knees extended and (trying to) put palms on the floor.

Both palms touch the floor

🞎 No

🞎 Yes

🞎 No contact with the floor

🞎 Fingertips touch the floor

🞎 Fingers touch the floor

🞎 Palms touch the floor

🞎 Wrists touch the floor

🞎 Forearms touch the floor

**Foot flexibility calcaneal stance position (3)**

Starting position: The participant stands groin width distance between feet.

Goniometer position and anatomical landmarks: Place the center of the goniometer on the middle of the calcaneus with the protractor of the goniometer facing up. Place the stationary arm of the goniometer on the floor. Place the movable arm of the goniometer along the longitudinal axis of the achilles tendon. Ninety degrees on the goniometer = 0 degrees. If the calcaneus is in a varus position, the measurement angle gets 0 degrees.

Stabilization: None


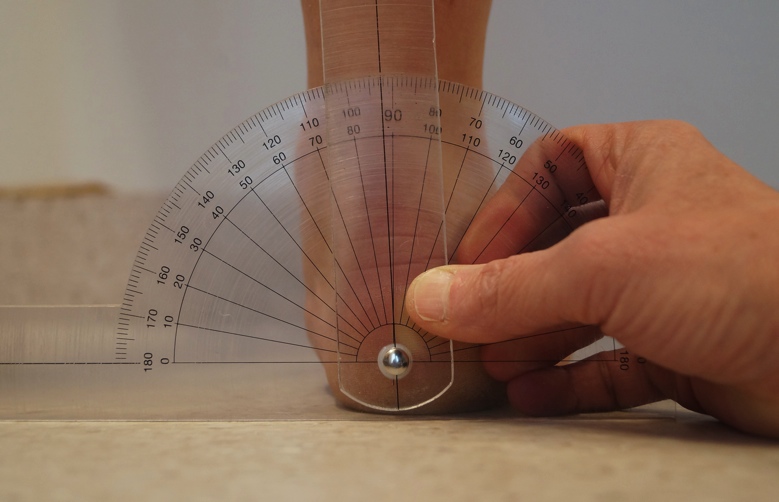
Performance: Standing.

Use a large goniometer.

Left calcaneus valgus:

🞎 No

🞎 Yes

Right calcaneus valgus:

🞎 No

🞎 Yes

Left calcaneus angle: _____

Right calcaneus angle: _____

**Active dorsiflexion + eversion (pronation) ankle (1, 4)**

Starting position: The participant stands half kneeling with hip and knee in 90 degrees and the hand against a chair to support balance. The foot to be measured is the front foot, fully
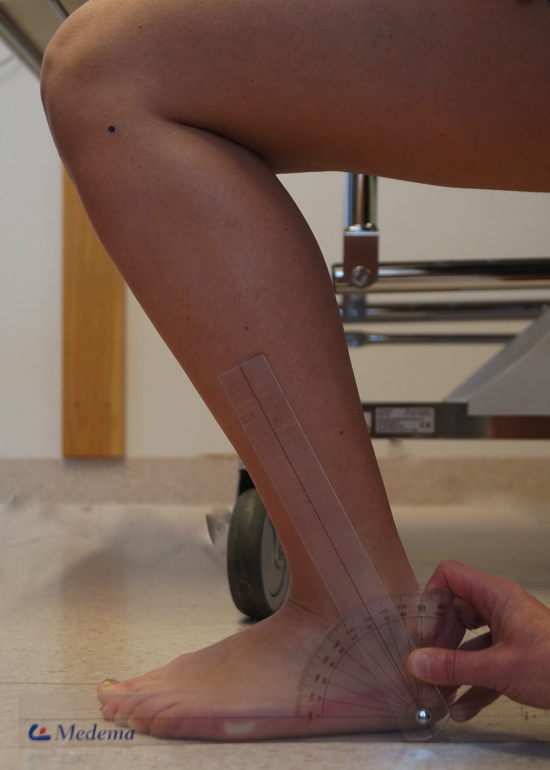
stabilized against the floor. The toes are pointing straight ahead. Mark reference dot on the head of the fibula.

Goniometer position and anatomical landmarks*:* Place the center of the goniometer inferior to the lateral malleolus. Place the stationary arm of the goniometer along the floor with the protractor of the goniometer facing up. Place the movable arm across the lateral malleolus, parallel to the longitudinal axis of the fibula toward its head and. Ninety degrees on the goniometer =0 degrees.

Stabilization: None

Performance: The participant actively flexes the knee and ankle as much as possible without lifting the heel from the floor. Use a large goniometer.

Left ankle angle: ______

Right ankle angle: ______

TESTS IN SUPINE POSITION

**Passive dorsal extension of the first metatarsophalangeal joint (1, 4)**

Starting position: The participant is in supine position with ankles and toes in neutral position. Mark a reference line along the first metatarsal.

Goniometer position and anatomical landmarks: Place the center of the goniometer on the medial side of the first metatarsophalangeal joint. Place the stationary arm of the goniometer parallel to the longitudinal axis of the first metatarsal. The movable arm is placed parallel to the longitudinal axis of the proximal phalanx along the first toe.

Stabilization: None


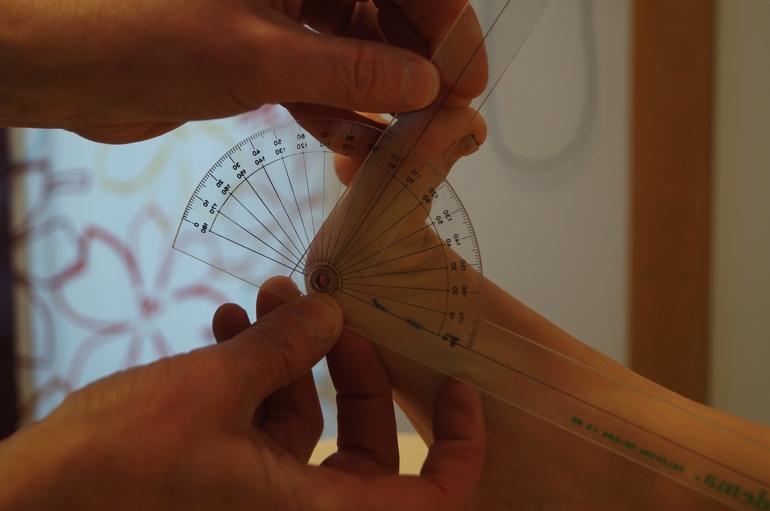
Performance: The assessor will slowly, passively push the first toe in further extension to ensure that the limit of motion is gained.

Use a small goniometer.

Left big toe angle: ______

Right big toe angle: ______

**Passive hyperextension of the knee (1, 2, 3, 4)**

Starting position: The participant is in supine position with knees straight. Mark reference dot on the greater trochanter.

Goniometer position and anatomical landmarks*:* Place the center of the goniometer over the lateral epicondyle of the femur with the protractor facing up. Place the stationary arm of the goniometer parallel to the longitudinal axis of femur pointing towards the greater trochanter and hold firmly. Place the movable arm of the goniometer parallel to the longitudinal axis of the fibula towards the lateral malleolus.

Stabilization: None

Performance: Place a heart level pillow (9x14 cm) under the heel with the flat side upwards with the knee hanging freely in the air. The participant will actively extend the knee to its maximum while the assessor places a hand above the knee and slowly presses the knee in passive maximum extension.

Use a large goniometer.


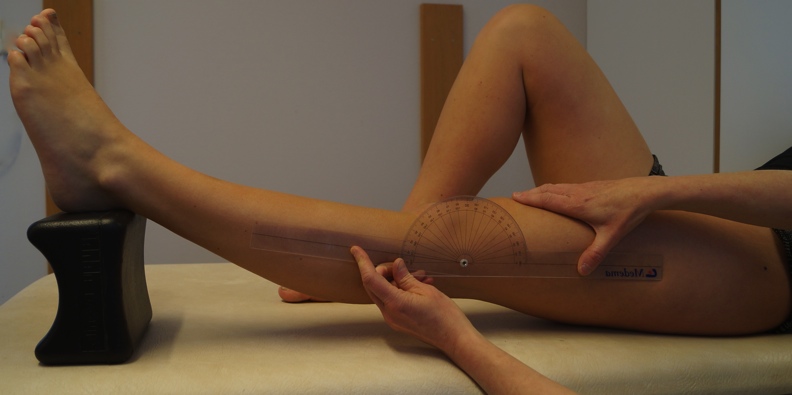


Left knee hyperextension:

🞎 No

🞎 Yes

Right knee hyperextension:

🞎 No

🞎 Yes

Left knee angle: ______

Right knee angle: ______

**Passive hip abduction (1, 4)**

Starting position: The participant is in supine position with straight knees and toes pointing toward the ceiling. Mark reference dots on sacroiliaca anterior superior (SIAS) and in the middle of patella.

Goniometer position and anatomical landmarks: Place the center of the goniometer over SIAS on the side being measured. The participant holds the stationary arm of the goniometer which is parallel to a straight line between the two SIAS. The movable arm of the goniometer is placed parallel to the longitudinal axis of the femur, pointing toward the middle of the patella.

Stabilization: None

Performance: The assessor moves slowly the leg passively into abduction. Make sure that no external rotation occurs in the hip and no movement occurs in the pelvis.


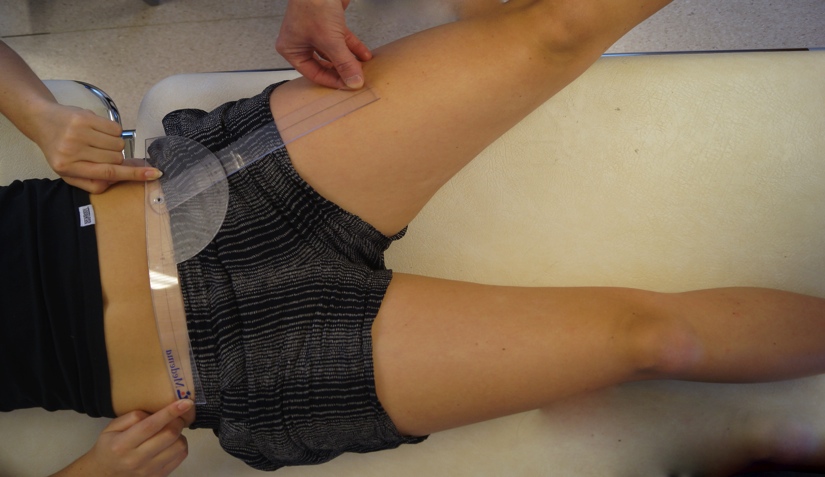
Use a large goniometer.

Left hip angle: ______

Right hip angle: ______

**Passive slide of the patella medial-lateral (1, 4)**

Starting position: The participant is in supine position with straight knees. Patella is divided into four quadrants by reference lines

Goniometer position and anatomical landmarks: None

Stabilization: None

Performance: The assessor slowly slides patella passively in medial direction using the thumb. Then slide patella in lateral direction using the index finger. Assess how many quadrants patella can slide in each direction.

Left patella: Medial Lateral

One quadrant 🞎 🞎

Two quadrants 🞎 🞎

Three quadrants 🞎 🞎

Four quadrants 🞎 🞎

Right patella:

One quadrant 🞎 🞎

Two quadrants 🞎 🞎

Three quadrants 🞎 🞎

Four quadrants 🞎 🞎


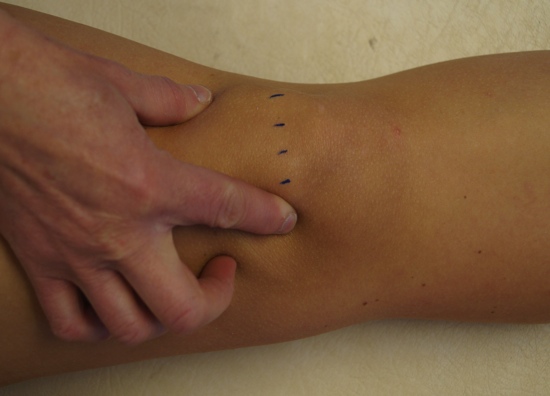

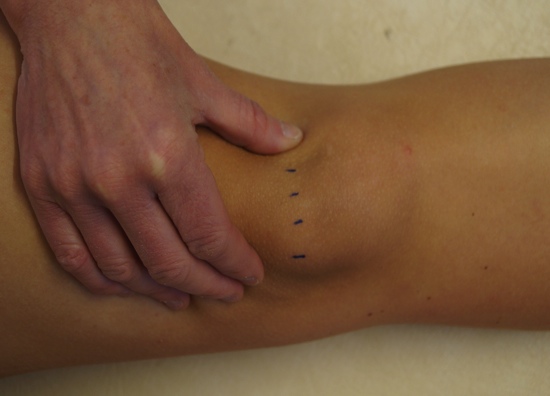


TESTS IN PRONE POSITION

**Passive flexion of the knee (4)**

Starting position: The participant is in prone position.

Goniometer position and anatomical landmarks*:* None

Stabilization: The assessor stabilizes the pelvis to prevent excessive extension in the lower back or flexion and abduction in the hip joint.

Performance: The participant passively flexes the knee with help of her hand around the ankle, moving the heel toward the buttock as far as she can.

Does the heel have contact with the buttocks?


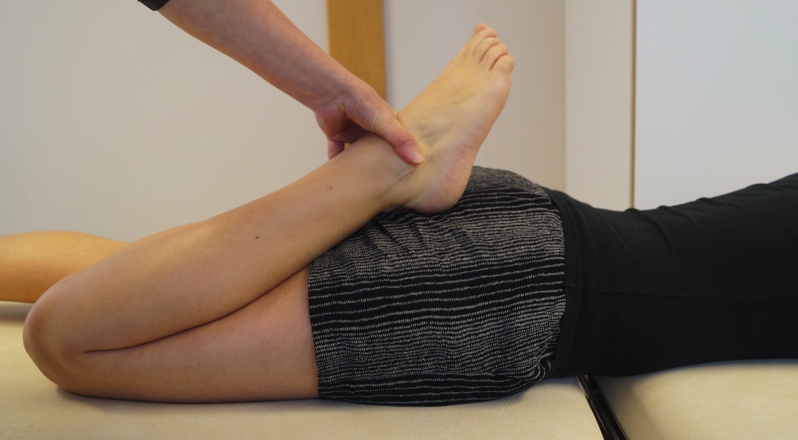


Left:

🞎No

🞎Yes

Right:

🞎No

🞎Yes

**Bruise**

Easy to get bruised after minimal trauma? (4)

🞎No

🞎Yes

**REMOVE ALL REFERENCE DOTS AFTER EACH ASSESSMENT**

**References**

1. Clarkson H. Musculosceletal Assessment, Joint Motion and Muscle Testing. Lippincott Williams & Wilkins. Third Ed, 2013
2. Beighton P, Solomon L, Soskolne CL. Articular mobility in an African population. Ann Rheum Dis 1973;32:413-418
3. McNerney JE, Johnston WB. Generalized ligamentous laxity, hallux abducto valgus and the first metatarsocuneiforme joint. J Am Podiatri Assoc 1979;69:69-82
4. Bulbena A, Duró J, Porta M, Faus S, Vallescar R, Martín-Santos R. Clinical assessment of hypermobility of joints:assembling criteria. J Rheumatol. 1992;19:115-122
5. Magee D J. Orthopedic Physical Assessment. Fifth edition 2008, 866-867. Saunders Elsevier
