# Supplementary material for: Inter- and intra-rater reliability for measurement of range of motion in joints included in three hypermobility assessment methods
Source: BMC Musculoskelet Disord. 2018 Oct 17;19:376. doi: 10.1186/s12891-018-2290-5 (PMC6192271; doi:10.1186/s12891-018-2290-5)
Supplement: Supplementary file 2 — Original description of the hypermobility instruments, the Beighton score, the Contompasis score and the Hospital del Mar Criteria. (DOCX 20 kb) [file 12891_2018_2290_MOESM2_ESM.docx]

Additional file 2

**Three assessment methods for generalized joint hypermobility (original versions)**

**The Beighton score (1)**

The subjects are given a numerical score of 0 to 9, one point being allocated for the ability to perform each of the following tests:

1. Passive dorsiflexion of the little fingers ≥ 90°
2. Passive apposition of the thumbs to the flexor aspects of the forearms
3. Hyperextension of the elbows ≥ 10°
4. Hyperextension of the knees ≥ 10°
5. Forward flexion of the trunk, with knees straight, so that the palms of the hands rested easily on the floor

Test 1-4 are performed bilaterally.

**The Contompasis score (2)**

Six tests are used and a numerical score is assigned to each test in accordance with the performance. Test 1-4 and test 6 are performed bilaterally.

1. Passive apposition of the thumb to the flexor aspect of the forearm (thumb to wrist test)

2 points: normal, 30 to 75 degrees, no touch of thumb to forearm,

4 points: low hypermobility, thumb touches forearm,

5 points: hypermobility, thumb digs into forearm easily

6 points: high hypermobility, thumb overlaps outside of forearm,

1. Passive dorsiflexion of the fifth metacarpophalangeal joint

2 points: normal, 30 to 85 degrees

4 points: low hypermobility, 90 to 100 degrees

5 points: hypermobility, 100 to 120 degrees

6 points: high hypermobility, 120 degrees and over

*Our modification:*

*2 points: 30-89 degrees*

*4 points: 90-99 degrees*

*6 points: 121 degrees and over*

1. Passive hyperextension of the elbow

2 points: normal, 0 to 5 degrees

4 points: low hypermobility, 10 to 16 degrees

5 points hypermobility, 16 to 20 degrees

6 points high hypermobility, 20 degrees and over

*Our modification:*

*2 points: 0-9 degrees*

*4 points: 10-15 degrees*

*6 points: 21 degrees and over*

1. Passive hyperextension of the knee

2 points: normal, 0 to 5 degrees

4 points: low hypermobility, 10 to 16 degrees

5 points: hypermobility, 16 to 20 degrees

6 points: high hypermobility, 20 degrees and over

*Our modification:*

*2 points: 0-9 degrees*

*4 points: 10-15 degrees*

*6 points: 21 degrees and over*

1. Hyperflexibility of the spinal column (forward flexion of the trunk)

2 points: normal, no contact with the ground

4 points: low hypermobility, fingertip touch to ground

5 points: hypermobility, fingers touching the ground

6 points: high hypermobility, palms to ground

7 or 8 points: highest hypermobility, wrist or forearm tog round

1. Foot flexibility test (ankle dorsiflexion and calcaneal stance position)

2 points: normal, 0 to 2 degrees eversion of calcaneus

4 points: low hypermobility, 3 to 5 degrees eversion of calcaneus

5 points: hypermobility, 6 to 10 degrees eversion of calcaneus

6 points: high hypermobility, 10 to 15 degrees eversion of calcaneus

8 points: highest hypermobility, 15 degrees and up eversion of calcaneus

*Our modification:*

*6 points: 11-15 degrees*

*8 points: 16 degrees and over*

A perfect score would be 22 points=no ligamentous laxity. This score is obtained by giving 2 points for a normal value in 5 tests bilaterally and 2 points for spine flexibility. The highest score would be 72 points.

**The Hospital del Mar criteria for the clinical assessment of joint hypermobility (3)**

A scale scoring 1 point/item, with an overall range 0 to 10, non-dominant side is tested.

One point being allocated for the ability to perform each of the following tests:

UPPER EXTREMITIES

1. Thumb: passive apposition of the thumb to the flexor aspect of the forearm at

< 21mm*.*

*Our* *modification: 21mm = 15 degrees* *(0 point >15, 1 point <15)*

1. Metacarpophalangeal: with the palm of the hand resting on the table, the passive dorsiflexion of the fifth finger is ≥ 90 degrees
2. Elbow hyperextension: the passive hyperextension of the elbow is ≥ 10 degrees
3. External shoulder rotation: with the upper arm touching the body and with the elbow flexed at 90 degrees, the forearm is taken in external rotation up to > 85 degrees of the sagital plane (shoulder to shoulder line)

LOWER EXTREMITIES, SUPINE POSITION

1. Hip abduction: the passive hip abduction can be taken to an angle of ≥ 85 degrees
2. Rotular (patella) hypermobility: holding with one hand the proximal end of the tibia, the rotula can be moved well to the sides with the other hand

*Our modification:*

*Left/right patella: Medial Lateral*

*One quadrant* 🞎 🞎

*Two quadrants* 🞎 🞎

*Three quadrants* 🞎 🞎

*Four quadrants* 🞎 🞎

1. Ankle and foot hypermobility: An excess range of passive dorsiflexion of the ankle and eversion of the foot can be produced.

*Our modification: 0 point <45 degrees, 1 point >45 degrees*

1. Metatarsophalangeal: Dorsiflexion of the toe of the foot over the diaphysis of the first metatarsal is ≥ 90 degrees

LOWER EXTREMITIES, PRONE POSITION

1. Knee hyperflexion: Knee flexion allows the heel to make contact with the buttock

-----------------------------------------------------------------------------------------------------------------

1. Ecchymosis *(easy bruising):* appearance of ecchymosis after hardly noticed, minimal traumatism

**References**

1. Beighton P, Solomon L, Soskolne CL. Articular mobility in an African population. Ann Rheum Dis 1973;32:413-418
2. McNerney JE, Johnston WB. Generalized ligamentous laxity, hallux abducto valgus and the first metatarsocuneiforme joint. J Am Podiatri Assoc 1979;69:69-82
3. Bulbena A, Duró J, Porta M, Faus S, Vallescar R, Martín-Santos R. Clinical assessment of hypermobility of joints: assembling criteria. J Rheumatol. 1992;19:115-122
